# An overview and a roadmap for artificial intelligence in hematology and oncology

**DOI:** 10.1007/s00432-023-04667-5

**Published:** 2023-03-15

**Authors:** Wiebke Rösler, Michael Altenbuchinger, Bettina Baeßler, Tim Beissbarth, Gernot Beutel, Robert Bock, Nikolas von Bubnoff, Jan-Niklas Eckardt, Sebastian Foersch, Chiara M. L. Loeffler, Jan Moritz Middeke, Martha-Lena Mueller, Thomas Oellerich, Benjamin Risse, André Scherag, Christoph Schliemann, Markus Scholz, Rainer Spang, Christian Thielscher, Ioannis Tsoukakis, Jakob Nikolas Kather

**Affiliations:** 1 Department for Medical Oncology and Hematology, University Hospital Zurich, Zurich, Switzerland; 2 Department of Medical Bioinformatics, University Medical Center Göttingen, Göttingen, Germany; 3 Department of Diagnostic and Interventional Radiology, University Hospital Würzburg, Würzburg, Germany; 4 Department for Hematology, Hemostasis, Oncology and Stem Cell Transplantation, Hannover Medical School, Hannover, Germany; 5 IMMS Institute for Microelectronics and Mechatronics Systems GmbH (NPO), Ilmenau, Germany; 6 Department of Hematology and Oncology, Medical Center, University of Schleswig Holstein, Campus Lübeck, Lübeck, Germany; 7 Department of Medicine 1, University Hospital Carl Gustav Carus, Technical University Dresden, Dresden, Germany; 8 Else Kroener Fresenius Center for Digital Health (EFFZ), Technical University Dresden, Dresden, Germany; 9 Institute of Pathology, University Medical Center Mainz, Mainz, Germany; 10 MLL Munich Leukemia Laboratory, Munich, Germany; 11 Medizinische Klinik 2-Haematology/Oncology, University Hospital, Frankfurt am Main, Germany; 12 Computer Vision and Machine Learning Systems Group, Institute for Geoinformatics, University of Münster, Münster, Germany; 13 Institute of Medical Statistics, Computer and Data Sciences, Jena University Hospital - Friedrich Schiller University, Jena, Germany; 14 Department of Medicine A, University Hospital Münster, Münster, Germany; 15 Institute for Medical Informatics, Statistics and Epidemiology, University of Leipzig, Leipzig, Germany; 16 Department of Statistical Bioinformatics, University of Regensburg, Regensburg, Germany; 17 Competence Center for Medical Economics, FOM University, Essen, Germany; 18 Department of Hematology and Oncology, Sana Klinikum Offenbach, Offenbach, Germany; 19 Medical Oncology, National Center for Tumor Diseases (NCT), University Hospital Heidelberg, Heidelberg, Germany

**Keywords:** Artificial intelligence, Machine learning, Digital health, Large language models, Computer vision

## Abstract

**Background:**

Artificial intelligence (AI) is influencing our society on many levels and has broad implications for the future practice of hematology and oncology. However, for many medical professionals and researchers, it often remains unclear what AI can and cannot do, and what are promising areas for a sensible application of AI in hematology and oncology. Finally, the limits and perils of using AI in oncology are not obvious to many healthcare professionals.

**Methods:**

In this article, we provide an expert-based consensus statement by the joint Working Group on “Artificial Intelligence in Hematology and Oncology” by the German Society of Hematology and Oncology (DGHO), the German Association for Medical Informatics, Biometry and Epidemiology (GMDS), and the Special Interest Group Digital Health of the German Informatics Society (GI). We provide a conceptual framework for AI in hematology and oncology.

**Results:**

First, we propose a technological definition, which we deliberately set in a narrow frame to mainly include the technical developments of the last ten years. Second, we present a taxonomy of clinically relevant AI systems, structured according to the type of clinical data they are used to analyze. Third, we show an overview of potential applications, including clinical, research, and educational environments with a focus on hematology and oncology.

**Conclusion:**

Thus, this article provides a point of reference for hematologists and oncologists, and at the same time sets forth a framework for the further development and clinical deployment of AI in hematology and oncology in the future.

## Introduction: The need for AI in hematology and oncology

Although Artificial intelligence (AI) is not a new research field (Schmidhuber 2022), recent developments driven by the availability of data sets and computing power, in particular, have led to the view that this rapidly evolving field may have the potential to transform our society (Rajpurkar et al. 2022). In the last ten years, AI has made significant advances in many different areas of society, including medicine. Hematology and oncology are a data-intensive and innovative medical specialty with a high clinical need for improved workflows and advanced methods for diagnosis and treatment guidance. Due to aging populations, cancer will become more prevalent in the next decades. At the same time, our capabilities to diagnose and treat cancer have multiplied in the recent past, and will continue to do so in the future. This creates a massively growing amount of data and an increasing complexity of clinical workflows. The complexity is further increased by advances in all medical specialties involved in treating cancer patients, including hematology and oncology, radiology, pathology, surgery, human genetics, nuclear medicine, and others. Patients′ heterogeneity in these regards require individualized solutions for which new scientific approaches need to be developed.

AI requires data to be available in a digital format. Digital data can be structured (such as data in a spreadsheet table or a database with pre-defined fields) or unstructured (such as unsegmented and/or non-annotated images or free text data). Many of these data types are routinely generated when diagnosing and treating cancer. Image data such as radiological or nuclear medicine imaging, as well as cytology or pathology images, are used to diagnose and stage tumors. Cancer subtyping is routinely performed using molecular and genetic testing, which can generate image data (for example, from fluorescence in situ hybridization or immunohistochemistry), genetic sequencing data (for example, genome, methylome, transcriptome data), metabolome data, proteome data, or other types. Aside from these data types, determining an optimal treatment strategy for a patient entails integrating a large number of highly variable pieces of information obtained from clinical examination, screening of previous medical records, and patient preferences. Moreover, general recommendations as derived, e.g., from randomized clinical trials are often not straight-forwardly transferable to every patient but require individual adaptations in the light of the individual patient characteristics.

In this setting, AI can be used for three purposes: (individualized) clinical care, research, and education. First, AI has the potential to be integrated into clinical routines and be used as a tool to aid humans in daily clinical practice. In this article, we mainly focus on this practical application of AI, since it can provide direct benefits to patients and physicians in hematology and oncology. For example, AI approaches and methods can be used to identify patterns in past cases that may help to predict how well a particular patient will respond to a specific treatment. Also, AI can assist to make treatment recommendations based on specific characteristics of each individual patient's tumor and monitor patients over time. In addition to this practical clinical use, there are two other aims. AI can be used as a research tool, allowing us to draw new scientific insights from clinical data such as new disease entities or pathomechanisms. In the future, it might be possible to better understand changes in the molecular-genetic and cellular composition of cancers, to derive new applications for existing drugs, to identify hidden patterns in oncological image data, to identify new therapy targets or pathologic processes, or to identify new biomarkers (Kleppe et al. 2021; Shmatko et al. 2022; Cifci et al. 2022). Finally, AI can be used as a tool for medical education, for example through the synthesis of data for educational purposes (Dolezal, et al. 2022; Krause et al. 2021; Chen et al. 2021). As populations age and cancer become more prevalent, more trained personnel are required to care for cancer patients. AI can potentially help to train these experts, although this aspect is still an emerging field in hematology and oncology.

To address, shape, and guide these advancements, the German Society of Hematology and Oncology (DGHO), the German Association for Medical Informatics, Biometry and Epidemiology (GMDS) and the Special Interest Group Digital Health of the German Informatics Society together established a joint working group “AI in Hematology and Oncology”, entrusted with serving as a central hub for all AI-related activity in the field of hematology, oncology and cancer research. The aim of this article, which represents the result of a collaborative effort within this group, is to provide a consensus definition of AI applications in hematology and oncology and to map out the most promising sub-fields for the near future.

## Definitions and terminology: what is AI in biomedicine?

**Fig. 1:**
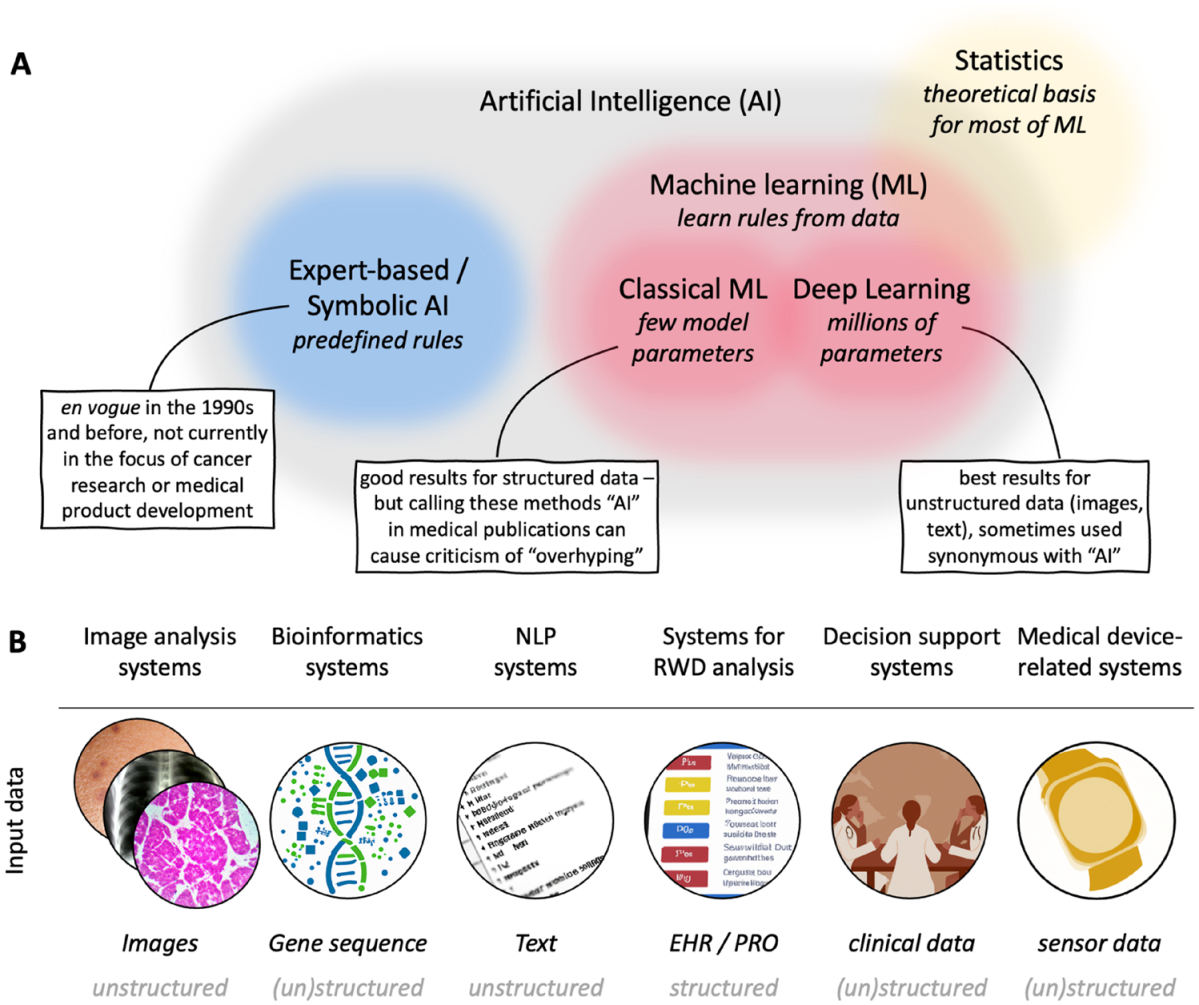
Artificial intelligence in oncology. **A** A simplified visualization of key methodological areas in oncological data science with example methods. **B** An expert-based definition of key fields of application of AI in oncology. Abbreviation: *NLP* natural language processing, *RWD* real-world data, *HER* electronic health record, *PRO* patient-reported outcomes

In the last decades, the field of AI has co-evolved with and drawn ideas from multiple adjacent research disciplines, and the terminology can be confusing (Fig. 1A). The terms machine learning (ML), deep learning (DL) and artificial intelligence (AI) have fuzzy boundaries which are heavily debated (Bzdok et al. 2018). In this article, we aim to provide a pragmatic definition of these methods, aiming to reflect the status quo in the biomedical research literature. Intuitively, a good example is as follows: The term "artificial intelligence" focuses on the word “intelligence”, implying that a machine performs some kind of intelligent service. The term "machine learning" focuses on the word “learning”, i.e., a machine learns something based on data with a certain aim, for example a disease model or a classifier. In a more formal, yet simplified view, two major sub-fields of AI have alternated and co-existed over the last decades: on the one hand, rule-based approaches, in which a human expert defines a fixed set of rules to classify data. This works well for very simple tasks based on structured data, but invariably fails for unstructured data and is also often not optimal for large quantities of structured data. On the other hand, ML, which does not encode any fixed rules, learns patterns from data. In the last five years, the ML paradigm within AI has made striking breakthroughs. In medicine, several ML-based algorithms have achieved regulatory approval in the last five years, while rule-based systems within AI are becoming less relevant to the practitioner (Shmatko et al. 2022; Benjamens et al. 2020). Hence, in this article, we will not discuss rule-based systems.

Within ML, there are three major classes of training strategies: reinforcement learning, supervised and unsupervised approaches (Shmatko et al. 2022). Reinforcement learning can help computer programs learn procedures, such as playing games, or navigating agents in a virtual world (Vinyals et al. 2019). Recently, reinforcement learning has been applied for medical applications, but it remains a niche approach in cancer research right now (Yala et al. 2022). Supervised techniques use labeled data to train a model, which then learns to predict the output based on the input. This is accomplished by feeding the model a series of input–output pairings known as training data, after which the model learns to maps the inputs to the matching outputs. This method is often used in problems including classification, regression, and prediction. Supervised techniques may be utilized in medical applications for tasks such as diagnosis, prognosis, and therapy planning. Unsupervised learning methods, on the other hand, do not use labeled data. Instead, the model is given a set of inputs and is expected to discover patterns or structure in the data on its own. Clustering, dimensionality reduction, and anomaly detection are examples of such tasks. Unsupervised techniques in medical applications can be used to identify subgroups within a patient population, find hidden patterns in medical imaging data, and detect aberrant patterns in datasets in general. Concerning clinical routine, supervised methods are more common, while unsupervised methods are sometimes used in medical research. In this article, we focus on supervised methods.

Within supervised ML, many different methods exist, and one way to distinguish them is by their model complexity, i.e., the number of trainable parameters. Most traditional ML methods which were used up until 2012 had dozens, hundreds or thousands of parameters per model. This includes a range of methods such as k-means clustering, support vector machines (SVMs), shallow neural networks, decision trees and random forests. Empirically, these methods are useful for structured data, such as tabular data, and to some degree for unstructured data, for example to classify image features which are extracted from radiology images according to pre-defined rules. Here, we refer to these methods as “Classical” ML. Since 2012, deep neural networks have emerged as a new tool with broad applications in medicine. Today’s deep neural networks build on mathematical theory, computer algorithms and hardware developed over many decades. Deep neural networks are different from “classical” ML methods in that they have millions or billions of parameters, and hence a much larger capacity to learn complex patterns. The use of deep neural networks is called “Deep Learning” (DL) and has become the dominant approach to process image, text, and other types of unstructured data in medicine. In the recent biomedical research literature, most “AI” studies are based on DL (Topol 2020, 2019). Medical applications of DL include diagnosis of disease, prediction of therapy outcomes, side effects or long-term prognosis.

## Application areas: How to use AI in hematology and oncology?

### Overview

AI methods can be used to address a broad range of clinical problems in hematology and oncology. In a consensus agreement of the AI working group, six classes of AI methods with established medical applications or potential clinical relevance were summarized (Fig. 1B). These methods are applicable to a broad range of clinical problems and can process a range of different data formats (Table 1). However, it should also be noted that there are AI approaches that can fall into several categories, e.g., when image and -omics data are jointly analyzed. In the following sections, each of these AI systems will be explained and examples for practical use cases will be given.

**Table 1:** An overview of AI systems in hematology and oncology with example applications

AI methods and applications	Source data (examples)	Clinical application in hematology and oncology (examples)
Image analysis systems	Radiology and nuclear medicine imaging data, histopathology image data, endoscopy, dermatoscopy, and others	aiding the diagnosis of tumors, assessing and predicting response to a given treatment, prognostication of the clinical course
Bioinformatics systems	Genetic sequencing data, and other -omics technologies	defining signatures of response to oncological treatments and targetable tumor subtypes
Natural language processing (NLP) systems	Spoken language or written notes (free text, unstructured data)	to automate the documentation or provide medical knowledge to doctors and patients, to extract data for further analysis from text, dialog systems (chatbots), analyze medical notes
Systems for real-world data (RWD) analysis	Electronic health records (EHR) and patient-reported outcomes (PRO)	Identification of adverse events, recommendation of treatment strategies
Decision support systems	Clinical and molecular data, data from multidisciplinary tumor boards, clinical data, time series	Recommendation of treatment strategies for a given patient
Medical-device related systems	Measurements from physical sensors, such as wearables and smart watches	(out)patient monitoring and prediction of treatment related complications

### Image analysis systems

The analysis of digital images is one of the most common applications of AI in oncology (Kleppe et al. 2021; Shmatko et al. 2022; Farina et al. 2022; Shreve et al. 2022; Luchini et al. 2022; Echle et al. 2021). In fact, most clinically approved algorithms using AI in medicine are related to image data (Benjamens et al. 2020; Muehlematter et al. 2021; Alexander et al. 2020). Images are often prone to subjective human interpretation, and especially reading medical imaging data requires years of training. Given sufficient training data, computer models have been shown to be able to perform on narrow tasks at the level of human experts (Shmatko et al. 2022; Shen et al. 2019; Nagendran et al. 2020; Tschandl, et al. 2020). For example, AI has been used successfully to diagnose diseases such as diabetic retinopathy (Natarajan et al. 2019; Sosale 2020; Quellec et al. 2021), melanoma (Balasubramaniam 2021; Brinker et al. 2019), or lung cancer (Jacobs et al. 2021; Ibrahim et al. 2021) from image data. In addition, AI may be able to detect features that are not immediately apparent to the naked eye. For example, the prognosis of lung cancer patients can be predicted from routine computer tomography image data. Traditionally, in the 2010s, “Radiomics” machine-learning methods have used sets of expert-defined visual “features”, coupled with simple ML models (Aerts et al. 2014). More recently, end-to-end Deep Learning has been increasingly applied to such tasks (Ghaffari Laleh et al. 2022).For example, AI has been used to prognosticate the course of colorectal cancer from digitized histopathology image data (Skrede et al. 2020), or to predict the response to immunotherapy from radiological imaging data (Trebeschi et al. 2019; Wu et al. 2019; Ligero et al. 2021). Also, AI has been used to predict the presence of genetic alterations from image data (Shmatko et al. 2022; Kockwelp, et al. 2022; Kather et al. 2020), and is being discussed as a potential way to pre-screen patients for targeted molecular testing (Shmatko et al. 2022). Thus, AI-based image analysis systems can serve two purposes in oncology: they can speed up diagnostic processes, make them more consistent and readily available even in low-resource settings. On the other hand, AI-based image analysis systems can in some circumstances extract prognostic or predictive information from images, and thus serve as a biomarker for precision oncology. In both types of applications—automation and biomarkers—rigorous clinical evidence is required before these systems are used broadly in clinical routine (Geis et al. 2019).

### Bioinformatics systems

Omics technologies (e.g., genomics, proteomics, metabolomics) generate large amounts of data that can be difficult for humans to interpret (Eraslan et al. 2019; Elmarakeby et al. 2021; Lipkova et al. 2022). In the last decades, computer-based methods to analyze these data have co-evolved with the laboratory assays (Shmatko et al. 2022). For example, the development of genome sequencing assays has been accompanied by the development of algorithms for sequence alignment and variant calling. Many bioinformatic machine-learning methods were developed for selecting molecular features and constructing molecular classifiers for disease entities or prognosis (Horn et al. 2018; Staiger et al. 2020). The development of all these methods has predated the era of Deep Learning. In fact, Deep Learning does not have a role in standard genetic diagnostics of cancer. However, additional useful information may be hiding in genome sequences and other -omics data. Several studies in the last few years also proposed Deep Learning methods to identify subtle patterns that were not identifiable by classical statistical approaches (Eraslan et al. 2019; Zeng et al. 2021; Tran et al. 2021). For example, AI has been used to predict outcomes and treatment response from sequencing data in cancer (Huang et al. 2020). Furthermore, -omics data might be combined with other data types (e. g., histopathology) to predict the clinical outcome of cancer patients more accurately (Chen et al. 2022). Researchers hope that by understanding genetic variants and their interplay in cellular networks in tumors they can find new therapeutic approaches, such as identifying potentially targetable neoantigens. Another field of application is the analysis of single-cell sequencing data often relying on neural network approaches such as variational auto-encoders. However, there is still a long way to go from academic research studies to embedding modern AI methods in clinical routine practice of genomically guided precision oncology.

### Natural language processing (NLP) systems

NLP is a branch of AI that deals with the interpretation and manipulation of human language (Yim et al. 2016; Sorin et al. 2020; Kung, et al. 2022; Yang et al. 2022; Singhal, et al. 2022). For example, NLP is used in chatbots and digital assistants such as Siri or Alexa, which are capable of understanding natural language commands and providing relevant information in response. In medicine, language is often used as an unstructured way to store and transmit information. In this context, NLP can be used to extract information from clinical reports or electronic health records (Yang et al. 2022; Thomas et al. 2014). It should be noted that current systems do not simply search and extract text but integrate the context (e.g., it is essential whether a diagnosis is present or excluded or how the temporal dependencies between reported events are). This information can then be used to support decision-making or generate predictions about disease progression or response to therapy. Although applications of NLP in oncology have been proposed more than five years ago (Yim et al. 2016), limitations of NLP methods have precluded widespread use. However, the research field of NLP is evolving rapidly and applications which were unimaginable as little as one or two years ago are now reality. Most recently, at the end of 2022, new large language models (LLMs) GPT-3 and its variant chatGPT by the company OpenAI have raised broad interest. Modern LLMs can converse like humans, can respond to questions in medical examinations (Kung, et al. 2022) and generally can be used as a search tool, in particular to answer medical questions. In the next few years, we expect an exponential increase in the application of NLP in oncology. Human-level NLP systems are just emerging and hence, the process to translate this technology to clinical value in oncology is also just beginning.

### Real-world data (RWD) analysis systems

Electronic health records (EHR) are at the core of documenting any patient contact in oncology, and also integrate multimodal data related to the diagnosis of cancer and biomarkers for precision oncology (Parikh et al. 2022; Morin et al. 2021; Araki et al. 2022). Much EHR data are unstructured or just loosely structured, making it difficult to mine historically. Moreover, such data is often distributed across different primary IT systems (e.g., laboratory information system or a hospital information system), which in turn may not be designed to support interoperability (the ability of a system to function effectively with other systems). AI methods have been applied to EHR data and promise to make the data available in a structured way and extract hidden value from the EHR data (Morin et al. 2021; Araki et al. 2022). Poor design of EHR is an unpleasant experience for many doctors and contributes to physician burnout (Muhiyaddin et al. 2022; Tajirian et al. 2020; Kroth et al. 2019). Thus, AI-based support systems to parse EHR data could be useful for users. EHRs often contain time series data which are challenging to analyze, but have been analyzed with neural networks or dynamical models (Kheifetz and Scholz 2019; Tomašev et al. 2019). While EHR comprises mostly data generated by healthcare staff, the patient perspective can be underrepresented. This gap is filled by patient-reported outcome- and experience measures (PROMs, PREMs) including a data source of the patient’s perspective which is increasingly being acknowledged in oncology as clinically relevant outcome measures in clinical trials and in certification processes evaluating health care in cancer centers (Parikh et al. 2022). EHR and PROM/PREM data are part of the loosely defined category of “real-world data” (RWD). We expect the AI-based analysis of RWD to be of much higher importance in the coming years, based on technological advances in multimodal AI models, NLP, and structured efforts to extract value from these data (Hegselmann, et al. 2022). The technology is now ready to be applied to many use cases, but progress in the field will be limited by asking the right medical questions, identifying useful ways to apply this technology in the clinic and the data quality of the original documentation.

### Decision support systems

Several AI systems have been developed to automate part of the complex decision-making process in oncology (Kheifetz and Scholz 2019; Schmidt 2017; Rodríguez Ruiz et al. 2022). Some of them use multidisciplinary tumor boards as a blueprint. The defining feature of such boards is that specialists from different disciplines (e.g., surgery, medical oncology, radiation oncology, radiology, pathology) come together to discuss individual patient cases and make treatment recommendations. The clinical history of a given patient, all available results from diagnostic tests, patient preferences and current medical evidence are integrated in this process (Frank 2022). The level of complexity in clinical decision-making in multidisciplinary tumor boards is increasing with the expanding significance of genomic and molecular data for personalized treatment recommendations in cancer care (Büttner et al. 2019; Horak et al. 2021). As early as 2012, large-scale and well-funded programs have aimed to automate such recommendations, but so far, they have not reached clinical routine due to the complexity of extracting standardized recommendations from inconsistent data (Schmidt 2017). Despite these experiences in the past, the use of AI to automate decision-making in a way analogous to multidisciplinary tumor boards is still being commonly mentioned as a promising application. (Rodríguez Ruiz et al. 2022)

### Medical hardware-related systems

For users, the boundaries between medical hardware devices and consumer devices are blurring more and more and wearable sensors are becoming more common. For example, smart watches are widely used nowadays and can collect a wide variety of data, including information on heart rate, oxygenation, and movement. AI algorithms may be able to make sense of these high-dimensional data and provide insights into a patient's health (Sabry et al. 2022). For example, wearable sensors have been used successfully to detect early signs of disease such as sepsis (Ghiasi 2022). In hematology, this could be used to identify patients at risk for severe side effects and impending organ failure, including patients after myelosuppressive chemotherapy or stem cell transplantation (Nessle et al. 2022). Despite the obvious potential in this area, clinical evidence is still scarce and only a few dozen published studies have investigated an application of AI-based analysis of wearable sensor data in oncology (Sabry et al. 2022; Ghiasi 2022). Often, such systems do not meet the criteria for regulatory approval as medical devices—especially if led by academic researchers. Again, this area will depend on hematologists and oncologists identifying the clinical need for new studies, running proof-of-concept studies and ultimately creating clinically relevant evidence how AI can be used for a patient benefit using properly designed clinical trials.

## Discussion and limitations

### Where are we headed?

In this article, we defined six main application areas in hematology and oncology where AI is already contributing or can contribute in the future. Some of these areas are already quite mature, for example, in AI-based image analysis, there are already dozens of approved medical devices that can be used in everyday clinical practice. Basic research or proof-of-concept work is still required in other areas, such as the support or partial automation of tumor board recommendations, modeling of individual time series data or the evaluation of sensor data with AI. Because of recent technological advances in AI, this technology is permeating our society more and more, and it is very likely that clinical management of patients in hematology and oncology will further benefit from AI approaches. As a result, it is critical that the transition to AI-assisted hematology and oncology is closely accompanied by a respective medical informatics infrastructure, new clinical trial concepts, and improved acceptance by doctors and patients.

### Data privacy and security

Whenever personal digital data are collected and linked together, the question of data privacy for the subject arises. It goes without saying that data in medicine must be securely stored, transferred, and protected from unauthorized access. This raises new issues in the age of artificial intelligence. Under certain conditions, it is possible to extract raw data from an AI network that has been trained on medical data and then published or sold for further use. In recent years, technological advancements such as differential privacy and secure multi-party computation have attempted to address this problem by introducing noise into the raw data of the training set or by privacy preserving computational approaches. However, in any case, it is critical that if possible, anonymized raw data are included from the start of the training process, and that, as is standard in medicine, an ethical approval and informed consent of the patient is available prior to the use and evaluation of patient-related data. (Seastedt et al. 2022)

### Biases, robustness and generalizability

Biases are another weakness of artificial intelligence and are particularly apparent in data-driven supervised machine-learning approaches (Andaur Navarro, et al. 2023). Machine-learning networks recapitulate and learn subtle patterns from training data, but these patterns are distorted in medicine and almost every other area of society due to prejudices and structural disadvantages for specific groups of people. If, for example, in the population represented in the training data, the implementation of complex molecular diagnostics is affected by place of residence, socio-economic factors, education, age, gender, or other factors, AI networks will learn these patterns on multiple levels and will eventually be able to apply them. Thus, use of an AI algorithm in the clinic may not generalize well enough to represent the diversity of future patients and therefore have an adverse effect on certain patient groups. The same is true if AI algorithms are trained and tested even on very large data sets from single institutions. Finally, high-parametric models are prone to overfitting, which reduces the performance in samples not used for model calibration. There is no perfect technical solution against such biases, but it is critical that both scientists and developers who set up the machine learning / AI system, as well as the end users, are made aware of them. Moreover, it should also be remembered that an appropriate study design is key to answer certain research questions (i.e., to evaluate the efficacy of an AI system, new concepts for randomized controlled trials will be needed). Further basic research in oncology is required to quantify the existence of such biases and to identify suitable areas of applications of these approaches and the potential harm to patients. It is precisely here that we see an important role for hematologists and oncologists, who, together with patients and patient advocacy groups, must ensure that such new technologies ultimately serve the well-being of patients and do not disadvantage specific patient groups based on their ethnicity, age, or other characteristics.

### Explainability and integration into clinical routines

Despite the possibilities of AI methods for hematology and oncology, these technologies often lack explainability since the underlying quantifications are based on highly complex calculations or learned network parameters which are not always directly relatable to biological mechanisms or structures. Despite the success of so-called explainable AI strategies (Minh et al. 2022), there are still many challenges, especially once these algorithms are used in critical situations such as clinical diagnoses and predictions (Ghassemi et al. 2021). Therefore and especially in the healthcare context, additional strategies have to be developed to enable informed clinical decisions. A possible approach could be to inform hypotheses-free neural network models by biological knowledge or by relating learned network structures or classifiers to biological quantities or risk factors. Integration of AI into clinical reasoning has to be done carefully, meaning that the computed results need to be treated as additional evidence helping clinicians to gain a more complete picture. We see this as an important area of fundamental research for the next few years.

### Digital literacy and AI literacy

We expect that physicians will be increasingly confronted with artificial intelligence applications in the future. (Mosch et al. 2022) It is, therefore, imperative that the ability to assess the outputs of such artificial intelligence systems is part of medical education and training. Even today, simple computer skills pose a challenge to some doctors, such as typing quickly on keyboards or the intuitive use of graphical user interfaces. In the future, the complexity of our world will continue to increase massively due to artificial intelligence, and that makes it necessary for doctors to continuously learn the required skills during their studies, and later, in their careers. We see a particularly important role here for medical professional societies, which will set up and implement the appropriate further training curriculum for doctors.

### Outlook

We expect that AI will be broadly used to aid clinical decision-making and improve the quality of care in the near future. While these technologies are maturing fast, the sensible clinical use of AI in hematology and oncology is determined by defining appropriate areas of application and by establishing the required IT infrastructures. Moreover, as for every new treatment concepts, AI-based approaches need to demonstrate their superiority in well-designed clinical trials. AI methods could result in disruptive changes in the clinical practice. For example, access to data may change, and treatment decisions may become more and more influenced by IT systems rather than practitioners. Thus, the way medicine is performed requires monitoring and guidance. In Germany, large-scale medical informatics initiatives have been proposed as platforms to facilitate data-intensive research with clinical routine data. However, on top of this, new clinical trial strategies are also required to prove the advantage of such AI-guided individual therapy concepts. In all of these efforts, patients, physicians and a network of experts in methodology should guide and lead the AI transformation of hematology and oncology and that professional medical societies such as the German Society for Hematology and Oncology (DGHO), the German Association for Medical Informatics, Biometry and Epidemiology (GMDS), and the German Informatics Society (GI), Special Interest Group Digital Health, together with other established initiatives and professional societies, will accompany and supervise this process.
